# Effects of Nitric Oxide on Neuromuscular Properties of Developing Zebrafish Embryos

**DOI:** 10.1371/journal.pone.0086930

**Published:** 2014-01-28

**Authors:** Michael Jay, Sophie Bradley, Jonathan Robert McDearmid

**Affiliations:** University of Leicester, Department of Biology, College of Medicine, Biological Sciences and Psychology, Leicester, United Kingdom; University Zürich, Switzerland

## Abstract

Nitric oxide is a bioactive signalling molecule that is known to affect a wide range of neurodevelopmental processes. However, its functional relevance to neuromuscular development is not fully understood. Here we have examined developmental roles of nitric oxide during formation and maturation of neuromuscular contacts in zebrafish. Using histochemical approaches we show that elevating nitric oxide levels reduces the number of neuromuscular synapses within the axial swimming muscles whilst inhibition of nitric oxide biosynthesis has the opposite effect. We further show that nitric oxide signalling does not change synapse density, suggesting that the observed effects are a consequence of previously reported changes in motor axon branch formation. Moreover, we have used *in vivo* patch clamp electrophysiology to examine the effects of nitric oxide on physiological maturation of zebrafish neuromuscular junctions. We show that developmental exposure to nitric oxide affects the kinetics of spontaneous miniature end plate currents and impacts the neuromuscular drive for locomotion. Taken together, our findings implicate nitrergic signalling in the regulation of zebrafish neuromuscular development and locomotor maturation.

## Introduction

Nitric oxide (NO) is a signalling molecule that regulates synaptogenesis in both central and peripheral nervous tissue [1]–[8]. This small, highly diffusible molecule, synthesized in biological tissues by a family of enzymes termed the nitric oxide synthases (NOS’s), mediates its effects principally via activation of soluble guanylyl cyclase (sGC) and cyclic guanosine monophosphate (cGMP) synthesis [9]–[11]. The NOS1 isozyme is expressed in neuronal tissue, often during periods of growth cone extension and synapse formation [1], [12]–[23] and can influence both synapse assembly [24]–[37] and maintenance [6], [8], [38], [39]. Recent evidence also suggests NO has developmental effects on the developing neuromuscular junction (NMJ): in *Xenopus* and chick embryos chronic NO treatment promotes acetylcholine (ACh) receptor clustering [2]–[5]. In addition, acute exposure to NO donors depresses spontaneous and evoked synaptic transmission at the NMJ of developing amphibians [40], an effect which may contribute to activity-dependent maturation of neuromuscular synapses. Moreover, work in our laboratory recently demonstrated that NO/cGMP signalling regulates arborisation of zebrafish spinal motoneurons. Here, NOS1 is observed in interneuron clusters that form near to motoneurons of the developing zebrafish spinal cord [22], [41]. Developmental inhibition of NO/cGMP activity markedly increases the number of collaterals formed on motor axons over the first three days of development whereas exogenous exposure to either NO donors or cGMP analogs has the opposite effect [41]. Whilst these observations strongly suggest that NO/cGMP signalling influences zebrafish motor axon development, the consequences to NMJ maturation remain poorly understood.

The aim of the current study was to determine how NO signalling influences anatomical and physiological maturation of zebrafish NMJs. Using histochemical approaches we show that developmental manipulation of NO and cGMP signalling affects the formation of NMJs along the axial swimming muscles of developing zebrafish. In addition, using *in vivo* patch clamp electrophysiology we show that developmental perturbation of NO affects the kinetics of spontaneous miniature end plate currents (mEPCs) at nascent NMJs. Finally, we provide evidence for NO-dependent effects on the maturation of locomotor network activity. Our data provides *in vivo* evidence that NO/cGMP signalling affects NMJ and locomotor maturation in zebrafish.

## Materials and Methods

### Ethics and Zebrafish Care

Zebrafish were maintained according to established procedures [42] and in compliance with the Animals (Scientific Procedures) Act 1986. Embryos were collected and incubated at 28.5°C in embryo medium until the required developmental stage. Staging was performed in accordance with Kimmel *et al*. [43]. All experiments were conducted on 2 day old zebrafish embryos. At this immature stage, zebrafish are not considered to be sentient and are unable to register pain. Thus ethics approval was not required for this study. On completion of electrophysiology experiments, embryos were anaesthetised with 0.02% MS-222 and killed with a bleach solution (sodium hypochlorite 6.15%). For histochemistry experiments, embryos were anaesthetised with 0.02% MS-222 and subsequently euthanised with 4% paraformaldehyde (PFA, Fisher Scientific).

### Pharmacological Reagents

During this study the following drugs were used: diethylenetriamine/nitric oxide adduct (DETA-NO, 250–500 µM, Sigma), *N*ω-Nitro-L-arginine methyl ester hydrochloride (L-NAME, 500 µM-1 mM, Sigma), 8-(4-Chlorophenylthio)-guanosine 3′, 5′-cyclic monophosphate sodium salt (8-pCPT-cGMP, 500–750 µM, Sigma), 1 H-[1], [2], [4]Oxadiazolo[4,3-a]quinoxalin-1-one (ODQ, 500 µM, Ascent Scientific), tetrodotoxin (TTX, 1 µM, Ascent Scientific), (+)-tubocurarine hydrochloride (tubocurarine, 3 µM, Sigma), formamide (2 M, Sigma) and 18-β-glycyrrhetinic acid (18βGA, 100 µM, Sigma).

### Chronic Drug Treatments

To exogenously elevate NO and cGMP levels the NO-donor DETA-NO and the cGMP analog 8-pCPT-cGMP were diluted in embryo medium (pH 7.4) to the desired concentration. Embryos at 24 hours post fertilisation (hpf) were placed in the resulting solution. Inhibition of NO or cGMP synthesis was achieved with L-NAME or ODQ, respectively. As these drugs appeared to exhibit low skin penetrance, they were dissolved in Evans physiological saline [composition (in mM): 134 NaCl, 2.9 KCl, 2.1 CaCl_2_, 1.2 MgCl2, and 10 HEPES, pH 7.8] containing fast green (0.2%; Sigma) and around 50 nl of the resulting solution was injected directly into the yolk. To do this, glass microinjection needles (tip diameter: 10–20 µm) were filled with drug solution and inserted into the yolk of 24 hpf embryos. Drugs were pressure-ejected using a Picospritzer microinjector (Parker, USA). Subsequent to drug treatment, embryos were incubated at 28.5°C until 48–52 hpf.

### Labelling of Putative NMJs

Histochemistry was performed as previously described [41]. Briefly, fish were anaesthetised in 0.02% MS-222 (Acros Organics) and subsequently fixed in 4% PFA for 90 min at room temperature. After thorough rinsing in PBSTX (Phosphate buffered saline containing 0.1% Triton-X 100), fish were placed in PBS containing 1 mg/ml collagenase (Sigma) for 16 min. After rinsing in PBSTX, fish were placed in blocking solution (composition: 3% milk powder; 1% DMSO; 0.1% Triton-X 100 in PBS) containing 10 µg/ml rhodamine-conjugated α-bungarotoxin (Rh-α-BTX; Sigma) which labels postsynaptic ACh receptors. After 30 min the block/Rh-α-BTX solution was rinsed off and fish were placed in blocking solution containing anti-SV2 antibodies (1∶200; Developmental Studies Hybridoma Bank (DSHB), University of Iowa), which label presynaptic neurotransmitter vesicles. Fish were incubated in primary antibody overnight at 4°C before thorough rinsing in PBSTX and incubation in fresh blocking solution. After 40 min AlexaFluor 488 secondary antibody (1∶500; Invitrogen) was added to the block solution and the fish were incubated overnight (4°C). Fish were subsequently rinsed with PBS and cleared in glycerol prior to mounting for image acquisition.

### Image Acquisition and Analysis of NMJ Distribution

**Figure 1:**
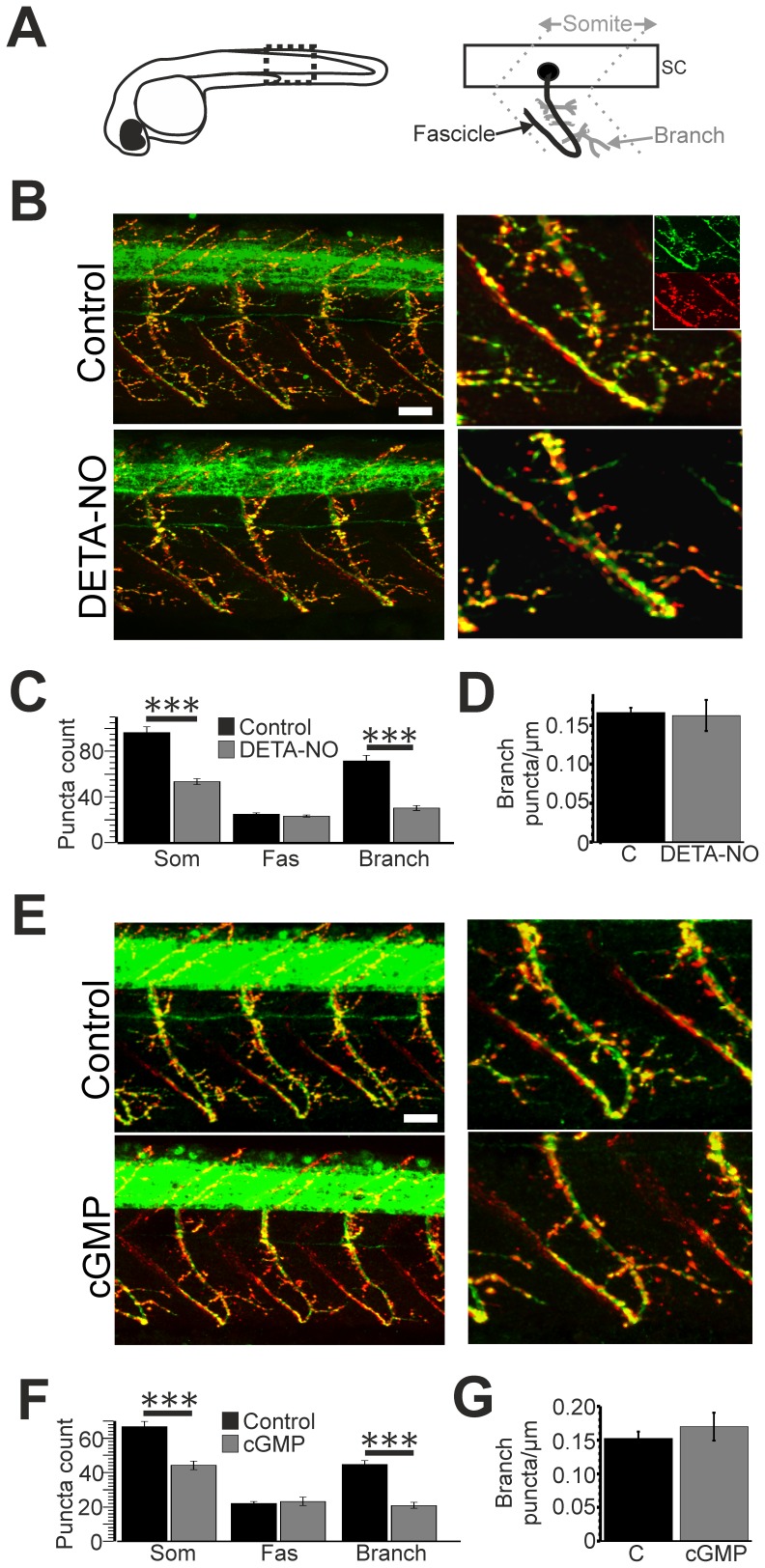
Developmental elevation of NO/cGMP signalling decreases NMJ numbers. **A**. Left: schematic illustration of a zebrafish embryo at 2 days post fertilisation (dpf). Dashed box indicates region used for puncta analysis. Right: schematic illustration of a motoneuron at 2 dpf. Motoneuron somata (black circle) are located within the spinal cord (sc) and their axons extend along fascicles (solid black line) into the somitic region. Axon branches (solid grey lines) extend from the main fascicle. Dashed lines indicate somitic boundaries **B.** Left hand panels: lateral trunk views of anti-SV2 (green)/Rh-α-BTX (red) co-staining in control (top) and DETA-NO treated (bottom) zebrafish at 2 dpf. Right hand panels: expanded regions showing staining localised to a single somitic region. Insets show SV2 (upper) and Rh-α-BTX (lower) z-projections from which merged images were derived. **C**. Bar chart depicting the mean (± SEM) number of synapses located within each somite (som), along motor fascicles (fas) and along branch-associated regions (branch) of control (black) and DETA-NO (grey) treated fish. **D**. Mean density of branch-associated puncta in control (black) and DETA-NO treated (grey) fish. **E.** Left hand panels: lateral trunk views of anti-SV2 (green)/Rh-α-BTX (red) co-staining in control (top) and 8-pCPT-cGMP treated (cGMP, bottom) zebrafish at 2 dpf. Right hand panels: expanded regions showing staining localised to a single somitic region. **F**. Bar chart depicting the mean (± SEM) number of synapses located within each somite (som), along motor fascicles (fas) and along branches of control (black) and 8-pCPT-cGMP (cGMP, grey) treated fish. **G.** Mean density of branch-associated puncta in control (black) and 8-pCPT-cGMP treated (cGMP, grey) fish. Scale bars = 30 µm. ***p≤0.001.

Labelled NMJs were imaged with an Olympus FV1000 confocal microscope connected to a PC running Fluoview FV1000 capture software. Images were captured using a 40x objective in z-stacks spaced at 3 µm increments [41]. Staining dorsal to the horizontal myoseptum was excluded from analysis. All experiments were conducted on fish ranging between 48 and 52 hpf and within each experimental replicate fish were carefully age-matched to minimise age-dependent variation. Images were obtained from trunk segments located at the level of the yolk-sac extension (Figure 1A). Acquired z-stacks were compressed into a single image and de-convoluted using Huygens Essential software prior to analysis with the ImageJ plugin SynaptcountJ. The accuracy of this method was confirmed via visual quantification of NMJ distribution. To analyse total NMJ number per somite, regions of interest (ROIs) were selected such that they spanned a single motor axon fascicle and its associated branches. For fascicle or branch analysis, ROIs were selected to encompass either the fascicular or motor axon branch domains within each somitic region. To quantify the density of branch-associated NMJs, the total number of branch-associated puncta was divided by the sum of axon branch lengths.

### In vivo Patch Clamp Electrophysiology

To prepare fish for physiological recording, 48–52 hpf embryos were anaesthetised in MS-222 (0.02%) and secured to a Sylgard-containing Petri dish via insertion of fine (0.025 mm) tungsten pins through the notochord. The trunk skin was carefully removed with a pair of fine forceps to expose the underlying muscle tissue. Fish were then transferred to a patch clamp microscope (Nikon FN-1) and the MS-222 washed off with Evans physiological saline containing 10 mM glucose. To gain access to embryonic fast twitch fibres, a small number of overlying embryonic slow twitch fibres were removed from a two to four somite region via aspiration with a broken-tipped patch electrode. All recordings were made from ventral fibres in somites adjacent to the tip of the yolk extension; approximately the same position used for imaging of NMJs (see above).

For all physiological experiments, whole cell patch clamp electrodes (resistance = 3–10 MΩ) were pulled from borosilicate glass (Harvard Apparatus, UK) using a P-80 micropipette puller (Sutter, USA). Electrodes were filled with a K-gluconate recording solution containing (in mM): 10 HEPES, 10 EGTA, 2 MgCl·6 H_2_O, 10 NaCl, 6 KCl and 126 D-gluconic acid-potassium salt (pH adjusted to 7.2 with KOH). During analysis of mEPCs, 1 µM TTX was added to the Evans physiological saline to ensure that recorded cells were synaptically isolated. For experiments in which mEPC kinetics were analysed, the gap junction blocker 18βGA (100 µM) was included in the bath solution in order to reduce contamination from electronically generated currents originating in neighbouring muscle fibres [44].

To study effects of acute DETA-NO/L-NAME application, fish were exposed to a 10–20 min pre-treatment with 2 M formamide prior to experimental recording. This treatment is known to cause effective excitation-contraction uncoupling [45], thus minimising the risk of muscle contraction during prolonged recordings. After 10 min in control saline, fish were exposed to a 10 min perfusion with either L-NAME or DETA-NO.

During analysis of locomotor-related end plate potentials (EPPs), a low concentration of the neuromuscular blocker tubocurarine (3 µM; Sigma) was substituted for TTX in the extracellular saline. Under these conditions muscle ACh receptors are partially blocked [46] such that muscular contractions are inhibited but locomotor-related drive to muscle fibres can be readily monitored with whole cell patch clamp electrophysiology. Fictive motor episodes were evoked with light stimulation.

During current clamp recordings muscle fibres were injected with a small amount of current to maintain a membrane potential of −75 mV and −65 mV for embryonic fast and slow twitch fibres respectively whereas during voltage clamp experiments, fibres were clamped at −75 mV. Fibres with access resistances >10 MΩ were routinely excluded and series resistance compensated by ≥70%. Biological signals were amplified using an RK-400 amplifier (Biologic) and digitized with a National Instruments A–D converter. Signals were acquired at a sample rate of 30 KHz and subsequently filtered to 2 kHz offline using a PC running pClamp (Molecular Devices).

### mEPC and EPP Analysis

All analysis was conducted offline in Clampfit (Molecular Devices). Events were detected using the template matching function and all captured events were subject to manual examination where erroneous events were excluded from analysis. mEPC frequency was determined by counting the number of events occurring over a 200 s recording period. Rise times (10–90%) and half-widths of mEPCs were analysed offline using Clampfit. For analysis of locomotor EPPs, 30 EPPs taken from each swim episode were captured using the template matching feature. These were pooled and averaged and the mean rise time (10–90%), decay time (10–90%) and amplitude recorded.

### Statistical Analysis

For analysis of NMJ puncta and mEPC frequency, statistical significance was determined using an unpaired Student’s *t*-test. Pairwise comparisons of mEPC kinetics were conducted using the Dunnett’s test and heteroscedasticity within mEPC datasets was controlled using the method described by Herberich *et al*. [47]. Results are presented as means ± standard error and statistical significance is reported as follows: *p<0.05; ** p<0.01; *** p<0.001.

## Results

### Developmental Effects of Elevated NO/cGMP Levels on NMJ Markers

Previously we had shown that NO signalling suppresses zebrafish motor axon branch development [41] and further demonstrated that chronic blockade of NOS (with L-NAME) increases the number of co-localised pre- and post-synaptic domains within the developing zebrafish musculature at 3 days post fertilisation (dpf). However, the effects of NO elevation and sGC/cGMP manipulation on NMJ formation have not been explored. Additionally, as we previously used voxel co-localisation methods to quantify changes in the number of volumetric pixels that contained overlapping synaptic markers, nitrergic effects on synapse number and distribution were not determined. To address these issues we manipulated NO/cGMP levels during development and labelled NMJs with the presynaptic marker anti-SV2 and the postsynaptic ACh receptor marker rhodamine conjugated α-bungarotoxin (Rh-α−BTX). Subsequently we examined the number and spatial distribution of NMJs with SynaptcountJ, a plugin for ImageJ that permits quantification of synapse number (see methods). Analysis was restricted to the second day of development (Figure 1A) because by this stage a small, easily quantifiable network of arbours has been established in each somitic region ([48]; Figure 1B). Since our previous work suggested that NO/cGMP signalling affects motor axon branch formation without impairing motor root growth, we segregated puncta into two domains: those located on the motor axon fascicle and those located on motor axon branches (Figure 1A). Using this approach, differential effects of NO/cGMP signalling on each compartment could be examined.

To characterise the effects of NO/cGMP signalling on neuromuscular synapse formation, 1 dpf embryos were exposed to embryo medium containing the NO donor DETA-NO or 8-pCPT-cGMP, a cell permeable cGMP analogue. Fish were incubated in drugs until 2 dpf, at which point they were processed for SV2/Rh-α-BTX histochemistry and compared to control fish raised in egg media alone. In agreement with previous reports [48], regions of SV2/Rh-α−BTX co-localisation (putative NMJs) were found scattered throughout the muscle of control embryos (Figure 1B). When developing embryos were exposed to DETA-NO, a marked decrease in the total number of somitic NMJs was observed (Figure 1B,C; control = 96.27±5.06, n = 11; DETA-NO = 53.33±2.54, n = 24, p<0.001). Analysis of puncta distribution revealed that this effect was caused by a reduction in branch-associated puncta (control = 71.45±4.87, n = 11; DETA-NO = 30.17±2.15, n = 24, p<0.001) whilst the number of fascicle-associated puncta was not affected (control = 24.82±1.17, n = 11; DETA-NO = 23.17±0.92, n = 24, p>0.05). This effect could arise from NO-dependent control of motor axon branch formation [41] or from a change in the number of NMJs formed per unit of branch length. To distinguish between these possibilities we calculated the density of branch-specific NMJ puncta, finding that DETA-NO had no effect on this parameter (Figure 1D; control = 0.17±0.01 puncta/µm, DETA-NO = 0.16±0.02 puncta/µm, p>0.05). This suggests that the NO-dependent reduction in NMJ number is a consequence of the previously reported reduction in motor axon branch formation [41].

Incubating embryos in 8-pCPT-cGMP had a similar effect to DETA-NO (Figure 1E,F) in that fewer NMJ puncta were observed (control = 66.80±2.96, n = 20; 8-pCPT-cGMP = 44.19±2.40, n = 16, p<0.001), an effect also caused by a reduction in branch-associated (control = 44.7±2.39, n = 20; 8-pCPT cGMP = 20.94±1.83, n = 16, p<0.001) but not fascicular (control = 22.1±1.043, n = 20; 8-pCPT-cGMP = 23.25±2.44, n = 16, p>0.05) puncta. Again, this observation could not be accounted for by a change in the density of branch-associated synapses (Figure 1G; control = 0.15±0.01 puncta/µm, 8-pCPT-cGMP = 0.17±0.01 puncta/µm, p>0.05). Collectively these observations suggest that developmental elevation of NO/cGMP reduces the formation of NMJs predominantly through suppression of motor axon branch formation.

### Developmental Effects of Inhibiting NOS/sGC on NMJ Markers

To determine the effects of reducing NO/cGMP levels during development we perturbed either NOS or sGC activity by exposing zebrafish embryos at 24 hpf to L-NAME or ODQ. Fish remained in drugs until 2 dpf, at which point they were processed for immunohistochemistry and compared to age-matched controls.

**Figure 2:**
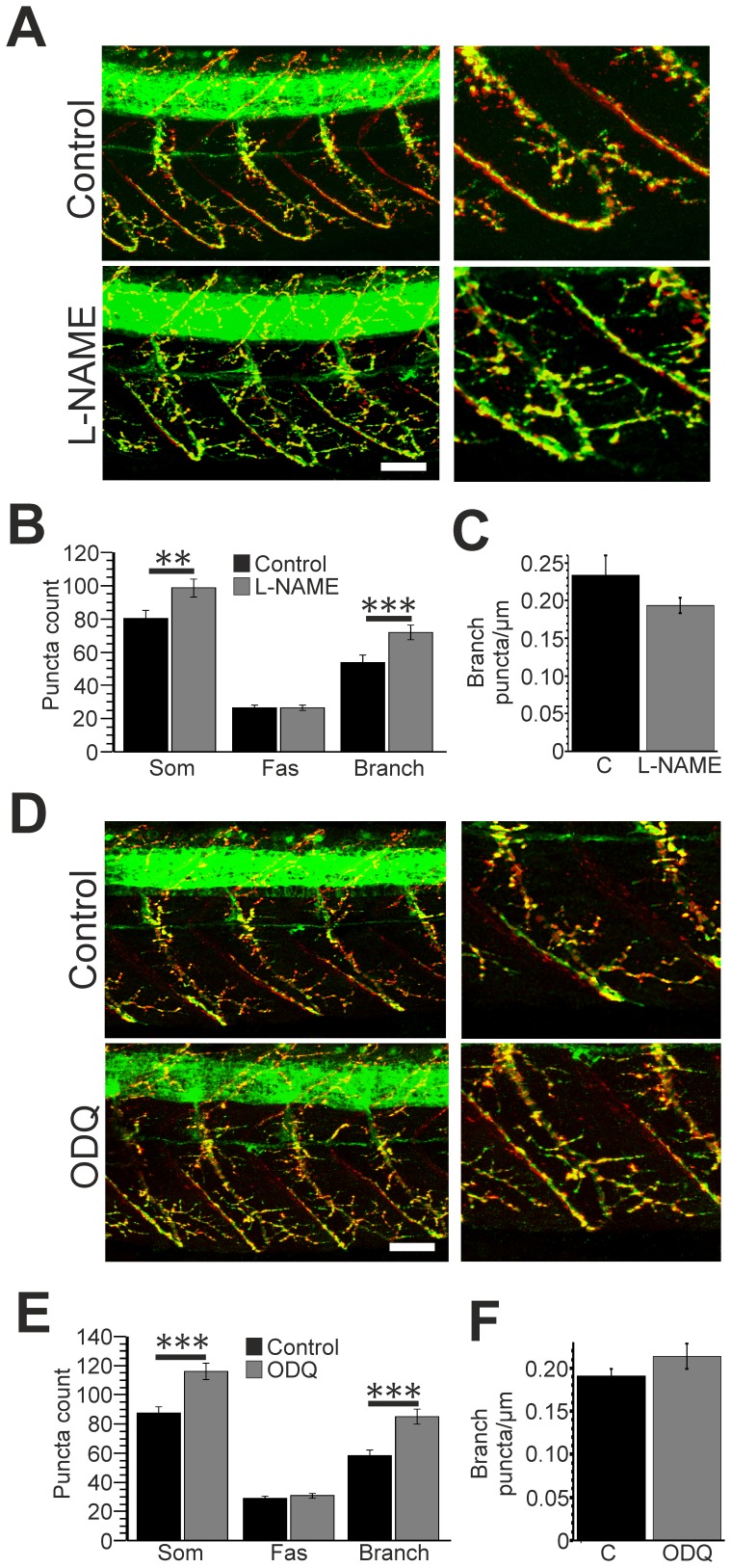
Developmental inhibition of NO/cGMP synthesis increases NMJ numbers. **A**. Left hand panels: lateral trunk views of control (top) and L-NAME (bottom) treated zebrafish at 2 days post fertilisation (dpf) processed with anti-SV2 (green) and Rh-α-BTX (red) staining. Right hand panels: expanded regions showing staining localised to a single somitic region. **B**. Bar chart depicting the mean (± SEM) number of synapses located within each somite (som), along motor fascicles (fas) and along branch-associated domains (branch) of control (black) and L-NAME (grey) treated fish. **C.** Mean density of branch-associated puncta in control (black) and L-NAME treated (grey) fish. **D**. Left hand panels: lateral trunk views of control (top) and ODQ (bottom) treated zebrafish at 2 dpf. Right hand panels: expanded regions showing staining localised to a single somitic region. **E**. Bar chart depicting the mean (± SEM) number of synapses located within each somite (som), along motor fascicles (fas) and along branch-associated domains (branch) of control (black) and ODQ (grey) treated fish. **F.** Mean density of branch-associated puncta in control (black) and ODQ treated (grey) fish. Scale bars = 30 µm. **p≤0.01, ***p≤0.001.

Developmental exposure to L-NAME (Figure 2A,B) significantly increased the number of somitic puncta (control = 80.30±4.77, n = 20; L-NAME = 98.56±5.27, n = 23, p<0.01). This was accompanied by an increase in branch-associated (control = 53.80±4.24, n = 20; L-NAME = 72.00±4.39, n = 23, p<0.001) but not fascicular (control = 26.50±1.75, n = 20; L-NAME = 26.56±1.47, n = 23, p>0.05) puncta. However, the density of branch-associated puncta was not affected by L-NAME treatment (Figure 2C; control = 0.23±0.03 puncta/µm, L-NAME = 0.19±0.01 puncta/µm, p>0.05). These findings are in agreement with previous observations showing that developmental exposure to L-NAME increases the number of co-localised NMJ markers at 3 dpf [41]. We also found that ODQ treatment (Figure 2D,E) increased somitic puncta number (control = 87.5±4.10, n = 18; ODQ = 116.00±5.67, n = 12, p<0.001), owing to an increase in branch-associated (control = 58.50±3.67, n = 18; ODQ = 85.17±3.67, n = 12, p<0.001) but not fascicular (control = 29.00±1.67, n = 18; ODQ = 30.83±1.70, n = 12, p>0.05) puncta. Again, we observed no effect on the density of branch-associated puncta (Figure 2F; control = 0.19±0.01 puncta/µm, ODQ = 0.21±0.01 puncta/µm, p>0.05).

### Developmental Effects of NO Signalling on mEPP Parameters

We next used electrophysiological approaches to determine whether developmental perturbations in NO signalling affected neuromuscular physiology. Two discreet muscle fibre populations, termed ‘embryonic fast’ (EF) and ‘embryonic slow’ (ES) are known to exist in early stage zebrafish [44], [49], [50]. These differ with respect to speed of contraction, position, orientation, extent of electrical coupling and synaptic kinetics: the ES fibres are superficial, run parallel to the body axis, are extensively electrically coupled to multiple neighbouring ES fibres and generate synaptic currents with relatively slow rise and decay kinetics [44]. By contrast, the EF fibres lie medial to the ES population, are arranged in an oblique orientation, exhibit less extensive electrical coupling and generate synaptic currents with relatively fast kinetics [44]. As a first step towards examining the effects of NO signalling on physiological maturation of zebrafish NMJs we examined the effects of NO on mEPCs occurring in both fibre populations. To this end we synaptically isolated muscle fibres of 2 dpf control, DETA-NO treated and L-NAME treated fish with the sodium channel blocker tetrodotoxin (TTX) and recorded mEPCs in the whole cell voltage clamp configuration [44], [49], [50].

**Figure 3:**
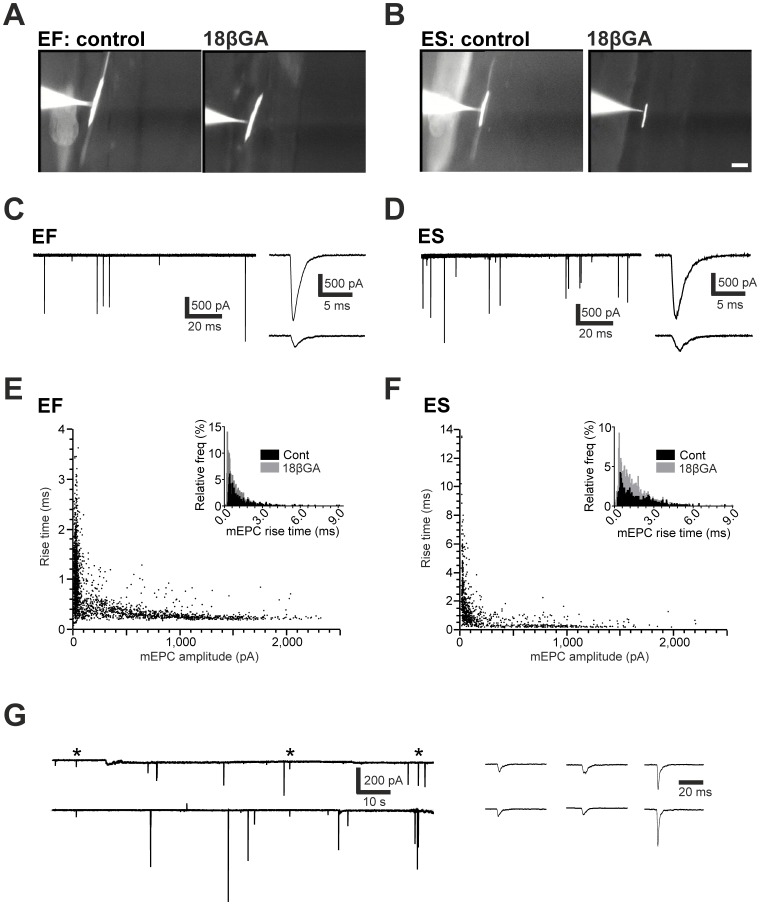
Effects of 18βGA on electrical coupling in EF and ES fibres. **A, B.** Images of embryonic fast (EF, **A**) and embryonic slow (ES, **B**) muscle fibres that were dialysed with sulforhodamine during whole cell recording. Note that in control saline, dye spreads to neighbouring muscle fibres whilst 18βGA pre-treatment abolishes this effect. **C,D.** Representative traces of miniature end plate currents (mEPCs) occurring in EF (**C**) and ES (**D**) fibres exposed to 18βGA. Right: example events captured on an expanded time scale. **E,F.** Amplitude versus rise time plots for mEPCs recorded from EF (**E**) and ES (**F**) fibres exposed to 18βGA. Insets show histograms of mEPC rise time in control (black) and 18βGA-treated (grey) fibres. **G.** Paired recording between ES fibres reveal that coincident events, presumed to be carried by electrical synapses, are occasionally observed in the presence of 18βGA; asterisked events with extended time scale on right. Scale bars = 50 µM.

During mEPC recordings, the gap junction blocker 18βGA was added to the extracellular saline in an attempt to block gap junction coupling between muscle fibres [44]. Electrical coupling has several effects on muscle physiology. First, it reduces fibre input resistance; second it decreases mEPC amplitude [44], [51] and; third, it introduces a sub-population of small amplitude, slow currents which are believed to be gap junction filtered representations of mEPCs occurring in neighbouring fibres [44], [50]. As expected, we found that 18βGA abolished transcellular dye labelling (Figure 3A,B), increased membrane resistance (control EF = 11.76±3.04 MΩ, 18βGA EF = 166.3±19.23 M Ω, p<0.001; control ES = 19.76±4.16 MΩ, 18βGA ES = 159.6±37.23 MΩ, p<0.01), increased mEPC amplitude (control EF = 73.16±2.92 pA, 18βGA EF = 783.80±14.22 pA, p<0.001; control ES = 55.58±1.72 pA, 18βGA ES = 626.10±19.86 pA, p<0.001) and dramatically reduced mEPC frequency (see below). However, we found that a proportion of small amplitude, slow currents persisted in the presence of this drug (Figure 3C,D). These were apparent in plots of mEPC rise time against amplitude where events segregated into two distinct populations, one with larger amplitudes and fast rise times and one with smaller amplitudes and slow rise times (Figure 3E,F). To determine whether the second population represented residual transjunctional events we recorded from pairs of adjacent muscle fibres (n = 1 EF pair, n = 3 ES pairs). We found that even after relatively prolonged (20–30 min) 18βGA exposure, coincident inward currents with small-amplitudes (<100 pA) and slow rise times (>0.6 ms) were observed in recorded muscle fibres (Figure 3G). Based on these observations, we considered these events to be mediated by passage of current through 18βGA insensitive gap junctions and thus excluded them from analysis.

**Figure 4:**
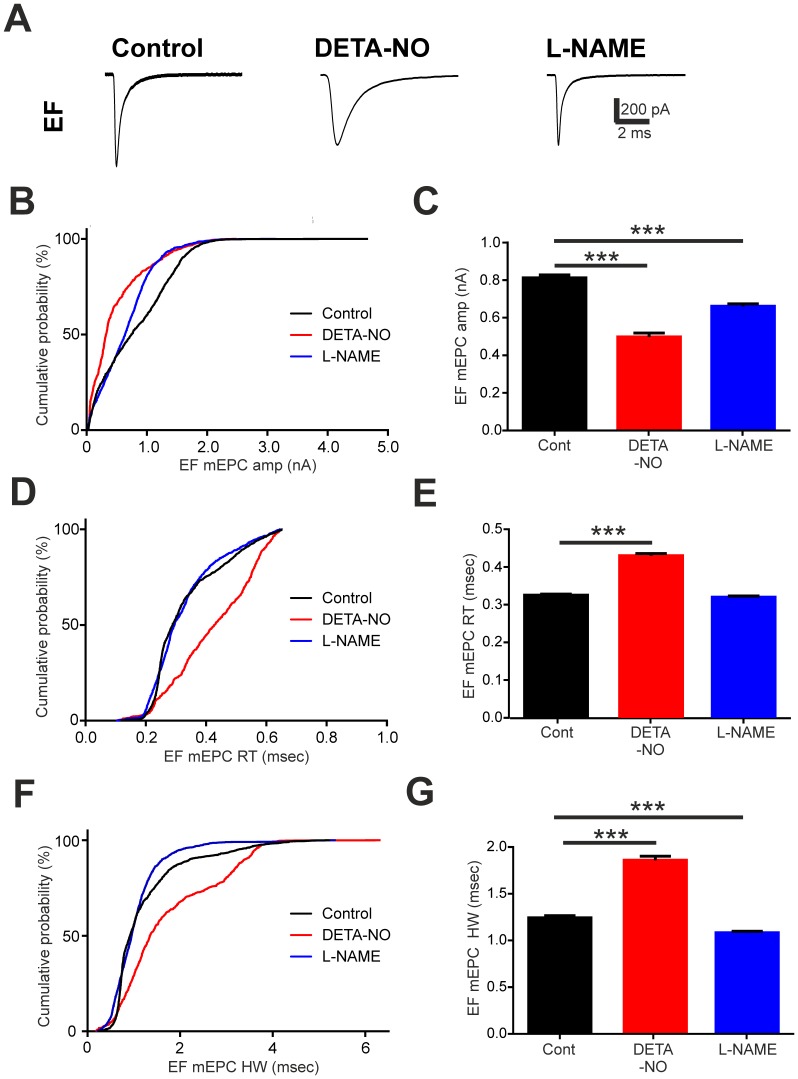
Developmental perturbation of NO signalling affects EF mEPC kinetics. **A.** Average traces of miniature end plate currents (mEPCs) captured during recordings of embryonic fast (EF) fibres in control (left), DETA-NO raised (middle) or L-NAME raised (right) fish at 2 days post fertilisation (dpf). **B–G**. Cumulative percentage plots and bar charts of EF mEPC amplitude (amp, **B,C**), rise time (RT, **D,E**) and half-width (HW, **F,G**) for each experimental condition. Data in **C,E,G** are represented as mean ± SEM. *** p<0.001.

We next compared mEPCs occurring in EF fibres of control, DETA-NO and L-NAME treated embryos (Figure 4A–G). Cumulative probability distributions indicated that developmental exposure to either DETA-NO or L-NAME reduced mEPC amplitude in EF fibres (Figure 4B) and analysis of mean amplitudes confirmed that this was the case (Figure 4C; control = 783.80±14.22 pA, n = 47 fibres; DETA-NO = 498.20±20.22 pA, n = 17 fibres; L-NAME = 661.40±13.01 pA, n = 46 fibres; p<0.001). Moreover, we observed a rightward shift in the cumulative distribution of EF mEPC rise times in DETA-NO treated fish (Figure 4D). This effect was accompanied by an increase in mean mEPC rise time (Figure 4E; control = 0.321±0.002 ms, n = 47 fibres; DETA-NO = 0.430±0.005 ms, n = 17 fibres; p<0.001). However, rise times were not significantly affected in L-NAME treated embryos (Figure 4D,E; control = 0.321±0.002 ms, n = 47 fibres; L-NAME = 0.320±0.003 ms, n = 46 fibres; p>0.05). Chronic manipulation of NO levels also affected decay kinetics of EF mEPCs (Figure 4F,G). Here, DETA-NO treatment caused a marked prolongation in mEPC half-width (control = 1.207±0.0.019 ms, n = 47 fibres; DETA-NO = 1.859±0.044 ms, n = 17 fibres; p<0.001) whereas L-NAME had the converse effect (control = 1.207±0.019 ms, n = 47 fibres; L-NAME = 1.083±0.003, n = 46 fibres; p<0.001).

**Figure 5:**
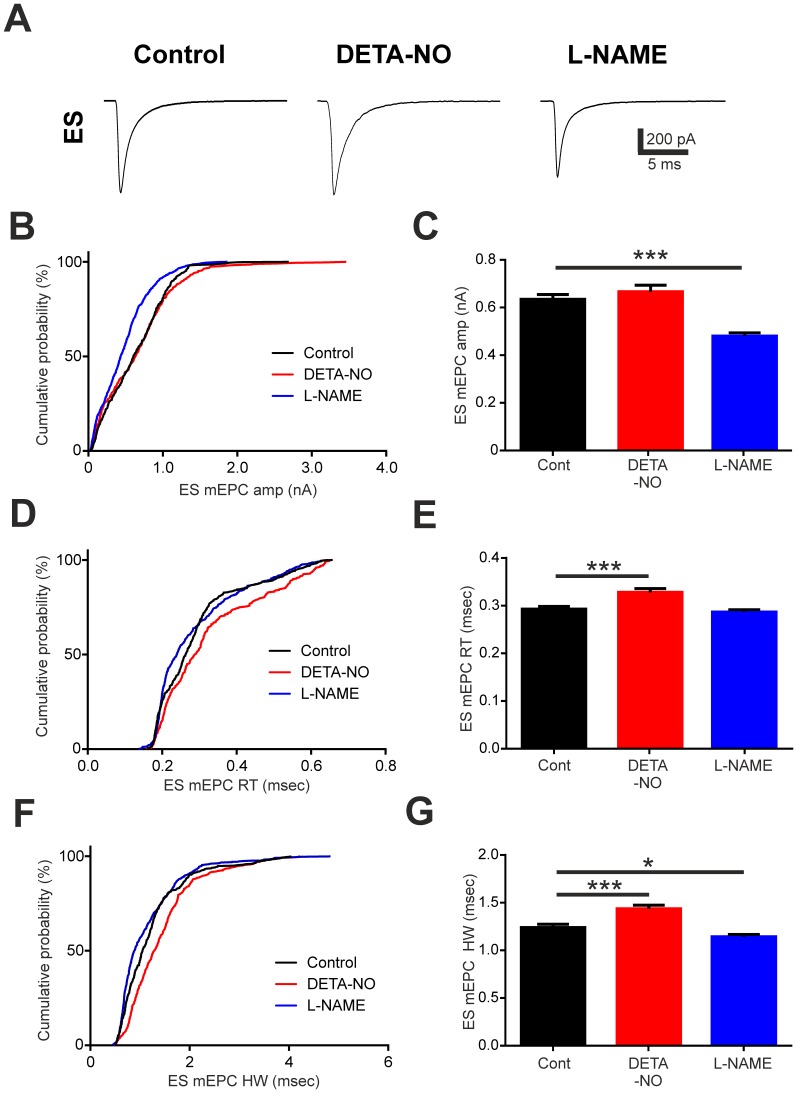
Developmental manipulation of NO affects ES mEPC kinetics. Average traces of miniature end plate currents (mEPCs) captured during voltage recordings in embryonic slow (ES) fibres of control (left), DETA-NO raised (middle) and L-NAME raised (right) fish. **B-G**. Cumulative percentage plots and bar charts of ES mEPC amplitude (amp, **B,C**), rise time (RT, **D,E**) and half-width (HW, **F,G**) for each experimental condition. Data in **C,E,G** are represented as mean ± SEM. * p<0.05, *** p<0.001.

Whole cell recordings of ES fibres revealed that NO signalling had similar, though less dramatic effects on mEPC parameters of these cells (Figure 5A–G). Here, ES mEPC amplitude (Figure 5B,C) was not affected by DETA-NO (control = 634.700±0.19.830 pA, n = 24 fibres; DETA = 661±27.330 pA, n = 33 fibres; p>0.05) but was significantly reduced in L-NAME treated fish (control = 634.700±0.19.830 pA, n = 24 fibres; L-NAME = 480.600±13.380 pA, n = 38 fibres; p<0.001). A significant increase in ES mEPC rise time (Figure 5D,E) was observed in DETA-NO treated fish (control = 0.293±0.006 ms, n = 24 fibres; DETA-NO = 0.328±0.007 ms, n = 33 fibres; p<0.001) whereas rise times in L-NAME treated fish were not significantly affected (control = 0.293±0.006 ms, n = 24 fibres; L-NAME = 0.287±0.005 ms, n = 38 fibres; p>0.05). Finally, the half-width of ES mEPCs (Figure 5F,G) was prolonged in DETA-NO treated fish (control = 1.239±0.034 ms, n = 24 fibres; DETA-NO = 1.435±0.039 ms, n = 33 fibres; p<0.001) and decreased in L-NAME treated fish (control = 1.239±0.034 ms, n = 24 fibres; L-NAME = 1.142±0.026 ms, n = 38 fibres; p<0.05). These observations are consistent with the hypothesis that developmental perturbation of NO signalling affects postsynaptic properties of EF and ES fibre NMJs.

Finally, we also examined mEPC frequency in EF and ES fibres. We found that in the presence of 18βGA, neither DETA-NO or L-NAME significantly affected the frequency of EF (control = 0.12±0.02 Hz, n = 47; DETA-NO = 0.10±0.03 Hz, n = 17; L-NAME = 0.11±0.02 Hz, n = 46, p>0.05, data not shown) or ES (control = 0.08±0.01 Hz, n = 24; DETA-NO = 0.06±0.01 Hz, n = 33; L-NAME = 0.08±0.01 Hz, n = 38; p>0.05, data not shown) mEPCs. However, during current clamp recordings in which 18βGA was excluded from the bathing media, DETA-NO decreased (control EF = 0.96±0.07 Hz, DETA-NO treated EF = 0.49±0.07 Hz; control ES = 1.26±0.09 Hz, DETA-NO treated ES = 0.95±0.09 Hz, p<0.05, data not shown) whereas L-NAME increased (control EF = 0.96±0.07 Hz, L-NAME treated EF = 1.24±0.10 Hz; control ES = 1.26±0.09 Hz, L-NAME treated ES = 1.71±0.14 Hz, p<0.01, data not shown) the number of synaptic events.

### Acute Manipulation of NO Levels does not Affect NMJ Physiology

**Figure 6:**
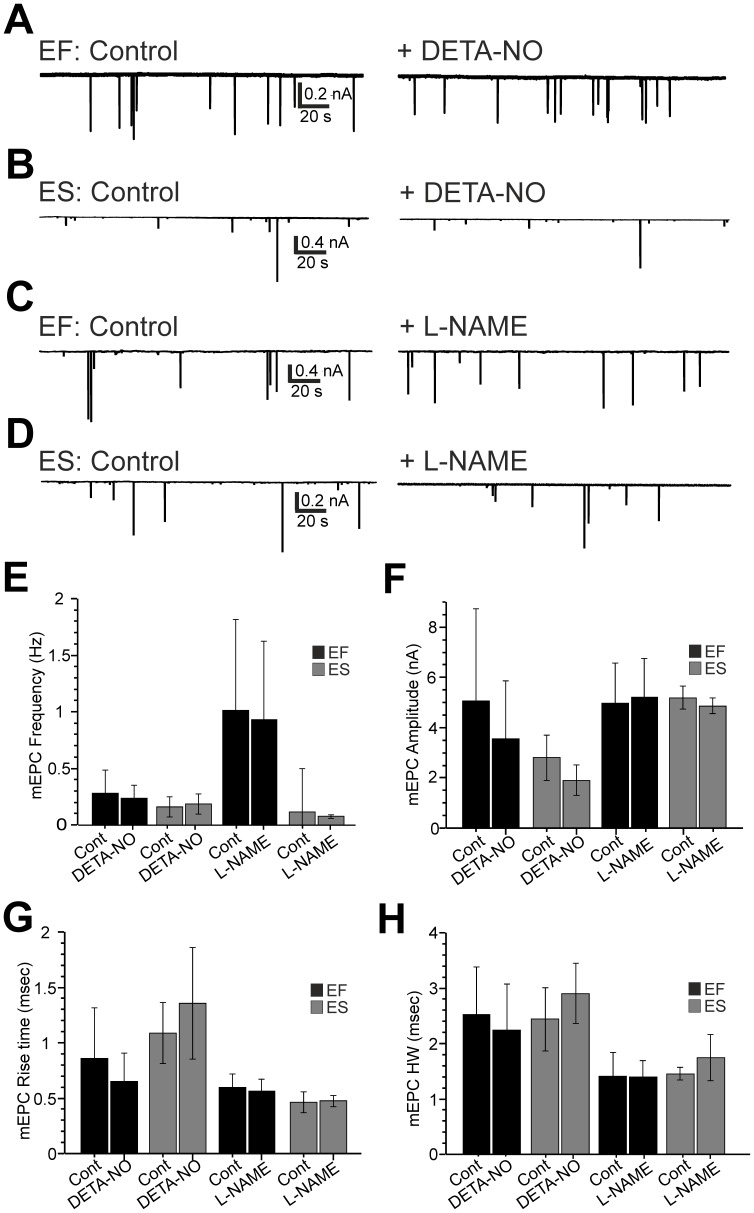
Acute manipulation of NO levels does not affect mEPC kinetics. **A–D**. Representative sweeps of embryonic fast (EF) and embryonic slow (ES) miniature end plate currents (mEPCs) in control conditions and after a 10 minute exposure to either DETA-NO (**A,B**) or L-NAME (**C,D**). **E–H**. Bar charts depicting effects of acute DETA-NO/L-NAME application on mean mEPC frequency (**E**), amplitude (**F**) rise time (**G**) and half-width (**H**). Data in E–H are represented as mean ± SEM.

To test for acute effects of NO signalling on NMJ physiology we obtained patch recordings from muscle fibres of 2 dpf control fish and subsequently exposed them to a brief (10 min) incubation in either DETA-NO (n = 3 EF, n = 3 ES) or L-NAME (n = 4 EF, n = 3 ES). During recordings (Figure 6A–D), neither bath perfusion of DETA-NO or L-NAME affected the frequency, amplitude, rise time or half-width of EF and ES mEPCs (Figure 6E–H). Thus, transient manipulation of NO signalling does not affect physiological parameters of the developing zebrafish NMJ.

### Developmental Effects of NO on the Locomotor Drive for Swimming

**Figure 7:**
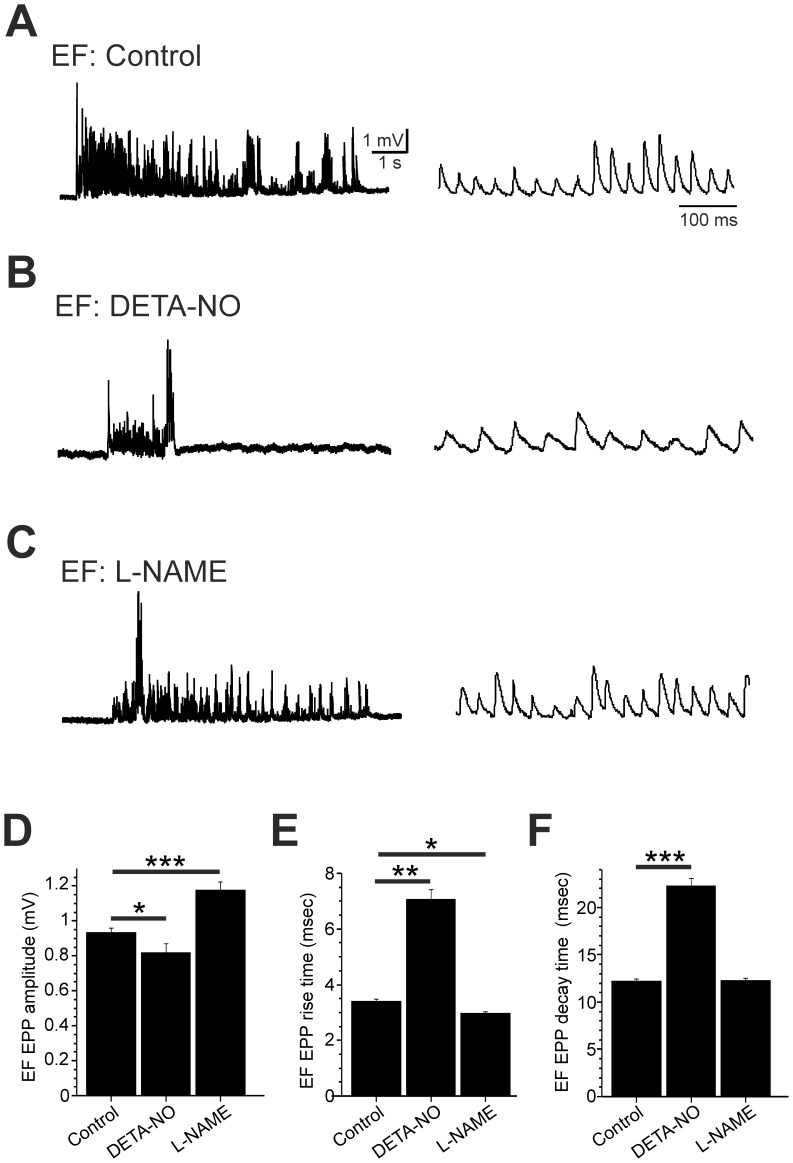
Developmental NO manipulation perturbs fictive locomotor drive to EF muscle fibres. **A–C**. Voltage recordings of locomotor-related drive obtained from embryonic fast (EF) fibres of 2 day post fertilisation (dpf) fish raised in control saline (**A**), DETA-NO (**B**) and L-NAME (**C**). **D–F**. Mean end plate potential (EPP) amplitude (**D**), rise time (**E**) and decay time (**F**) measured during episodes of fictive swimming. Data in **D–F** are represented as mean ± SEM. * p<0.05, ** p<0.01, *** p<0.001.

**Figure 8:**
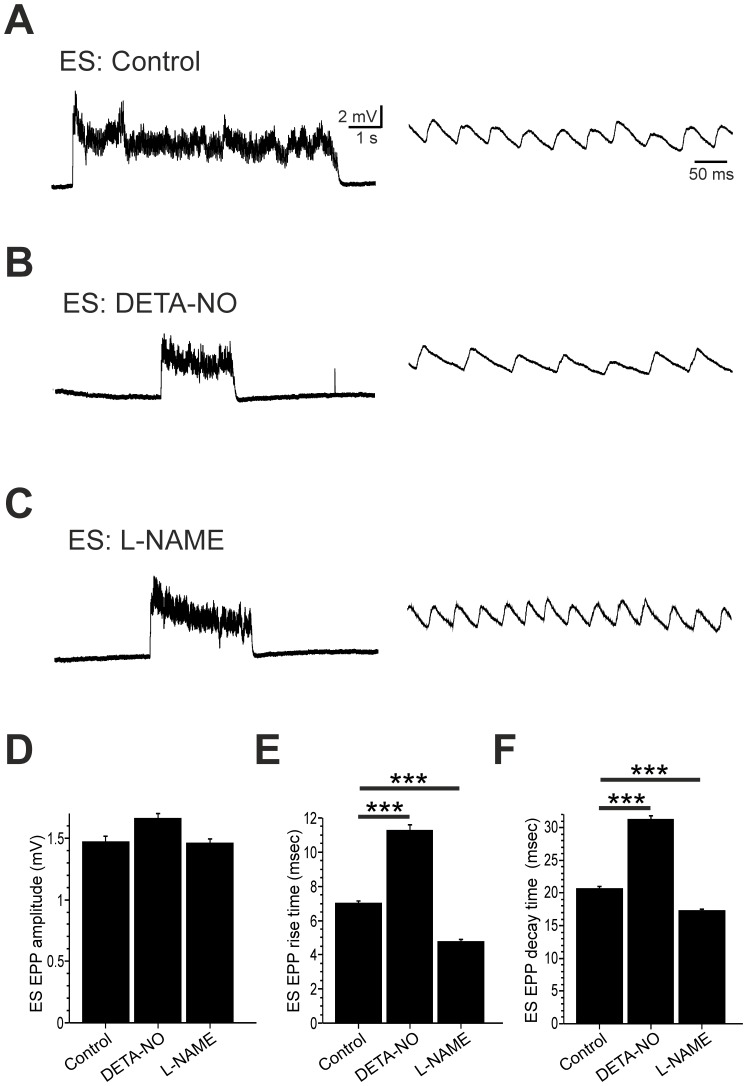
Developmental NO manipulation perturbs fictive locomotor drive to ES muscle fibres. **A–C.** Voltage recordings of locomotor-related drive obtained from embryonic slow (ES) fibres of control (**A**), DETA-NO raised (**B**) and L-NAME raised (**C**) fish at 2 days post fertilisation (dpf). **D–F**. Mean end plate potential (EPP) amplitude (**D**), rise time (**E**) and decay time (**F**) measured during episodes of fictive swimming. Data in **D-F** are represented as mean ± SEM. *** p<0.001.

We next asked how developmental perturbation of NO affected the neuromuscular drive for swimming behaviour. To do this we examined locomotor-related end plate potentials (EPPs) in EF (Figure 7) and ES (Figure 8) fibres that were not exposed to 18βGA. Using this approach we were thus able to study the NO-dependent regulation of locomotor ontogeny under more physiologically-relevant conditions. The synaptic drive for locomotion was monitored during sensory-evoked bouts of fictive swimming in 2 dpf fish raised in control saline (n = 10 EF; n = 10 ES), DETA-NO (n = 4 EF; n = 4 ES) or L-NAME (n = 7 EF; n = 9 ES). During EF recordings, developmental DETA-NO exposure (Figure 7A,B) decreased locomotor EPP amplitudes (Figure 7D; control = 0.94±0.03 mV; DETA-NO = 0.82±0.05 mV, p<0.05) and prolonged EPP rise (Figure 7E; control = 3.40±0.08 ms; DETA-NO = 7.07±0.36 ms, p<0.01) and decay (Figure 7F; control = 12.21±0.08 ms; DETA-NO = 22.24±0.80 ms, p<0.001) durations. In contrast, L-NAME (Figure 7A,C) increased locomotor EPP amplitudes (Figure 7D; control = 0.94±0.03 mV; L-NAME = 1.17±0.04 mV, p<0.001) and induced a small, yet significant decrease in EPP rise times (Figure 7E; control = 3.40±0.08 ms; L-NAME = 2.96±0.08 ms, p<0.05) but did not affect decay times (Figure 7F). During ES recordings (Figure 8A–C), neither pharmacological reagent affected the amplitude of locomotor-related EPPs (Figure 8D). However, marked effects on EPP rise and decay times were observed. Here, DETA-NO dramatically increased EPP rise (Figure 8E; control = 7.02±0.13 ms; DETA-NO = 11.27±0.32 ms, p<0.001) and decay (Figure 8F; control = 20.66±0.29 ms; DETA-NO = 31.29±05.2 ms, p<0.001) durations whereas L-NAME raised fish exhibited a significant decrease in rise (Figure 8E; control = 7.02±0.13 ms; L-NAME = 4.80±0.11 ms, p<0.001) and decay (Figure 8F; control = 20.66±0.29 ms; L-NAME = 17.30±0.27 ms, p<0.001) times.

**Figure 9:**
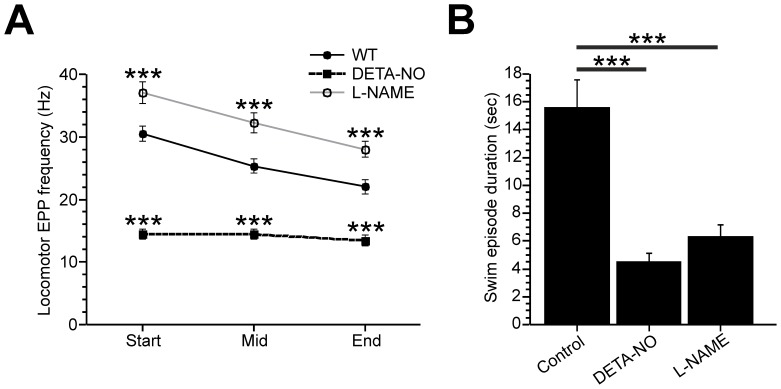
Developmental effects of NO on the frequency and duration on the neuromuscular drive for locomotion. **A**. Line graph of mean (± SEM) locomotor-related end plate potential (EPP) frequency at the beginning, middle and end of evoked fictive swim episodes in control fish and fish exposed to DETA-NO or L-NAME during development. **B**. Bar chart showing mean duration of fictive motor episodes in control, DETA-NO and L-NAME treated fish. *** p<0.001.

To determine how NO affected the frequency and duration of motor episodes, data from EF and ES recordings were pooled. Thereafter locomotor frequency was estimated by measuring EPP frequency during the beginning, middle and end of each episode. When compared to controls, EPP frequency of DETA-NO exposed fish was consistently lower across all periods of the locomotor episode (Figure 9A; p<0.001). By contrast, mean EPP frequency was higher throughout episodes of L-NAME treated fish (Figure 9A; p<0.001). In addition, developmental exposure to both DETA-NO and L-NAME caused a significant reduction in the duration of locomotor episodes (Figure 9B; control = 15.6±1.98 s; DETA-NO = 4.53±0.60 s; L-NAME = 6.34±0.90 s, p<0.001). Taken together, these data strongly suggest that developmental manipulation of NO signalling impacts locomotor performance.

## Discussion

The main findings of this study are that developmental manipulation of NO signalling reduces the number of neuromuscular synapses and affects the physiological properties of NMJs within the developing muscle population of zebrafish embryos. When taken together, our observations provide *in vivo* evidence that endogenous NO signalling influences the formation and functional maturation of zebrafish NMJs.

### NO/cGMP Signalling as a Regulator of Neuromuscular Synaptogenesis

Our previous study revealed that NO signalling suppresses and NOS inhibition enhances motor axon branch development in zebrafish. Moreover, voxel analysis revealed that the number of co-localised pre- and post-synaptic NMJ markers is increased by chronic L-NAME exposure. In the current study we have built upon these findings so that the effects of NO and cGMP on NMJ number, density and distribution could be determined. This allowed us to make three important observations. First, we show that developmental elevation of NO/cGMP (via addition of DETA-NO/8-pCPT-cGMP) inhibits, whilst developmental inhibition of NO/cGMP synthesis (via exposure to L-NAME/ODQ) promotes, formation of NMJs within the axial swimming muscles. Second, we show that these effects arise from changes in the number of puncta located on motor axon branches, but not fascicles. Finally, we show that the density of branch associated-synapses is not affected by NO signalling. Since our previous data demonstrates that NO decreases motor axon branch number [41], we posit that the NO-dependent modifications in NMJ number reported here arise as a consequence of modified branch formation. However, once branches are formed, NO does not appear to alter the rate of synapse addition.

During the current study we found that NO signalling did not affect the number of fascicular NMJs. This is perhaps not surprising as drug treatment was initiated at 24 hpf. By this stage, early periods of motor fascicle extension and the incorporation of pre-patterned ACh receptors into fascicular NMJs is complete [48], [52], [53]. Whilst we cannot exclude the possibility that pharmacological treatment at earlier time points may influence fascicular synaptogenesis, the observation that NOS1 is first expressed within the spinal cord at around 30 hpf [41] strongly suggests that NO has no physiological role during initial stages of neuromuscular development which begins at ∼ 17 hpf [54]. Thus, unlike amphibian and avian species, where NO affects aneural ACh receptor cluster assembly [2]–[5], the earliest stages of zebrafish NMJ development are unlikely to be NO-dependent.

### NO Signalling and NMJ Physiology

During mEPC experiments we routinely included 18βGA in the bath solution to block electrical synapses within the zebrafish musculature. This drug has previously been reported to abolish the low-amplitude, filtered events that arise from electrotonic spread of current from electrically coupled neighbouring fibres [44]. Here, we found that 18βGA application abolished transcellular spread of sulforhodamine and increased both muscle fibre input resistance and mEPC amplitude. Although these observations suggest that 18βGA abolished gap junction signalling, we found a population of small, filtered mEPCs persisted in the presence of this drug. Interestingly, Sylvain *et al.* [55] reported a similar phenomenon following exposure to the 18βGA analog carbenoxolone, reasoning that they were generated by nascent synapses located on the recorded muscle fibre. However, as we found that similar events often occurred synchronously during paired muscle recordings, we favour the hypothesis that they are caused by current flow through 18βGA-resistant gap junctions. Thus, for the purposes of our study, this population of events was excluded from analysis.

Our data suggests that chronic manipulation of NO signalling had marked effects on synaptically-mediated mEPCs. Specifically, developmental elevation of NO (DETA-NO exposure) caused a marked slowing of mEPC rise times and half-widths in EF and ES muscle. By contrast, mEPC half-widths of L-NAME treated fish were reduced in EF and ES fibres, although rise times were not significantly affected. In addition, L-NAME reduced the amplitude of EF and ES mEPCs whilst DETA-NO reduced EF mEPC amplitudes. Collectively, these observations suggest that NO may regulate the maturation of immature neuromuscular synapses. In support of this hypothesis, previous studies have shown that the kinetics of zebrafish mEPPs and mEPCs change markedly during neuromuscular ontogeny. During early embryonic development, these exhibit prolonged rise and decay times and relatively small amplitudes. As maturation proceeds, rise and decay times shorten whilst amplitude increases [46], [50], [56], [57]. The speeding-up of synaptic currents has been attributed to a combination of changes that include maturation of presynaptic properties, the gradual aggregation and addition of ACh receptors at the endplate and the expression of ACh-esterase, which serves to hydrolyse ACh within the synaptic cleft and thus accelerates relaxation of endplate currents [57]–[59]. In addition, vertebrate neuromuscular ACh receptors often undergo developmentally-related changes in subunit composition which engenders differing kinetic properties during development. For example, immature ACh receptors are typically composed of α2βδγ subunits whilst adult receptors contain α2βδε subunits [60]–[63]. Receptors expressing the γ subunit exhibit prolonged open channel times and generate prolonged macroscopic currents whereas those expressing the ε subunit exhibit short open channel times and generate shorter macroscopic currents [61]. Whilst a delay in the switch from γ to ε subunits may contribute to the effects of NO on EF fibres, it is unlikely to explain the effects observed within ES fibres as they do not exhibit a developmentally-related change in subunit composition [59]. Future molecular and biochemical studies are needed to identify whether one or more of these developmental phenomena are influenced by NO signalling.

Interestingly, we found that the NO-dependent effects on mEPC frequency varied as a function of recording conditions. In the absence of 18βGA, event frequency decreased in DETA-NO treated fish but increased in L-NAME treated fish. When considered in light of our anatomical findings, we hypothesise that these effects are caused by NO-dependent regulation of NMJ numbers. By contrast, differences in mEPC frequency were not observed when 18βGA was included in the extracellular saline. These effects could arise because differences in mEPC frequency may only be apparent when inputs to multiple muscle fibres are simultaneously monitored. Addition of 18βGA would reduce electrical coupling between fibres, thus reducing the number of fibres from which events could be sampled.

### Developmental Effects of NO on Locomotor Drive

Although transient NO biosynthesis is known to modulate parameters of ongoing locomotor activity [64], [65], its relevance to locomotor maturation has remained unclear. Here we show that chronic manipulation of NO signalling can strongly affect locomotor network output. Developmental exposure to DETA-NO prolongs the rise and decay durations of locomotor-related EPPs. Whilst the effects of NOS inhibition (L-NAME incubation) were less pronounced, we did observe a small acceleration in EF/ES EPP rise times and a reduction in ES EPP decay times during locomotor activity. Given that mEPC rise and decay times are prolonged in DETA-NO and accelerated in L-NAME, the observed changes to the neuromuscular drive may, at least in part, arise from a change in the waveform of macroscopic currents generated by ACh receptor activation. Specifically, DETA-NO dependent slowing of the rise and decay time of ACh receptor currents would be expected to slow rates of membrane depolarisations and repolarisations during swimming. By contrast, L-NAME, which accelerated the rise and decay times of mEPCs would be expected to have the opposite effect. However, other factors may also contribute to this phenomenon. For example, the extensive electrical coupling between zebrafish embryo muscle fibres is known to filter postsynaptic responses within the muscle [44], [50], [66]. Thus, a change in the extent of electrical coupling could affect the rise and decay times of EPPs recorded during swimming. Additionally, the amplitude, rise and decay time of compound EPPs is known to be influenced by the time course of transmitter release from individual presynaptic terminals [67], [68]. A number of factors, including synaptic ACh [69], catecholamines [70] and presynaptic action potential duration [68], [71] have been shown to influence release latency at the NMJ and as a consequence affect the rise, decay and amplitude of postsynaptic events. On this note, NO has been reported to directly affect ACh release at the NMJ [72], catecholamine release within the spinal cord [65] and neuronal excitability of interneuron populations [73] and so could potentially alter release latency via a number of different mechanisms. Future motoneuron-muscle recordings will help to determine whether NO does indeed have an effect on the timecourse of evoked ACh release at the developing NMJ.

We also found that when compared to controls, the frequency of locomotor-related EPPs was lower in DETA-NO treated and higher in L-NAME treated fish, an observation that stands in broad agreement with our previous behavioural studies [41]. In addition, we observed that both DETA-NO and L-NAME exposure reduced the duration of locomotor-related episodes. Although the mechanism of action awaits investigation, this effect is likely to be caused by an NO-dependent effect upon the properties of spinal cord interneurons or descending motor control pathways, rather than NO-dependent regulation of NMJ properties: at the onset of swimming (approximately 26 hpf), the locomotor network contains a small number of immature neurons that can only generate low-frequency, inflexible forms of swimming behaviour [46], [74]. As electrical properties of spinal neurons mature and new cells are integrated into the spinal network, fish become capable of swimming at a wide range of frequencies [75]–[80]. Thus, an NO-dependent effect on the development of later-developing spinal interneurons may underlie the observed actions of NO. Alternatively, observed changes in motor output might arise from homeostatic feedback mechanisms that scale the frequency of rhythm generator activity to the biophysical characteristics of the muscle. Thus, the NO-dependent slowing of postsynaptic NMJ kinetics may trigger feedback mechanisms that ensure network activity is limited to frequencies that do not cause EPP summation and tetanus. In contrast, the speeding up of postsynaptic responses in conditions of low NO may allow the locomotor network to drive muscles at slightly higher frequencies. Irrespective of the causes, our observations stand in agreement with previous behavioural studies showing a reduction in swim frequency in fish exposed to NO donors during development and an acceleration in locomotor output in fish lacking the NOS1 isozyme [41].

In summary, our data provide strong evidence that NO serves as a developmental regulator of NMJ maturation in zebrafish. These observations provide further support for the hypothesis that NO is a fundamentally important signalling molecule during periods of NMJ synaptogenesis and locomotor maturation.

## References

[1] Roskams AJ, Bredt DS, Dawson TM, Ronnett GV (1994) Nitric oxide mediates the formation of synaptic connections in developing and regenerating olfactory receptor neurons. Neuron 13: 289–299. 7520251 10.1016/0896-6273(94)90347-6

[2] Schwarte RC, Godfrey EW (2004) Nitric oxide synthase activity is required for postsynaptic differentiation of the embryonic neuromuscular junction. Dev Biol 273: 276–284. 15328012 10.1016/j.ydbio.2004.06.003

[3] Jones MA, Werle MJ (2000) Nitric oxide is a downstream mediator of agrin-induced acetylcholine receptor aggregation. Mol Cell Neurosci 16: 649–660. 11083925 10.1006/mcne.2000.0901

[4] Godfrey EW, Schwarte RC (2003) The role of nitric oxide signaling in the formation of the neuromuscular junction. J Neurocytol 32: 591–602. 15034255 10.1023/B:NEUR.0000020612.87729.98

[5] Godfrey EW, Longacher M, Neiswender H, Schwarte RC, Browning DD (2007) Guanylate cyclase and cyclic GMP-dependent protein kinase regulate agrin signaling at the developing neuromuscular junction. Dev Biol 307: 195–201. 17560564 10.1016/j.ydbio.2007.04.021 PMC1978166

[6] Cogen J, Cohen-Cory S (2000) Nitric oxide modulates retinal ganglion cell axon arbor remodeling in vivo. J Neurobiol 45: 120–133. 11018773 10.1002/1097-4695(20001105)45:2<120::aid-neu6>3.0.co;2-6

[7] Wu J, Fang L, Lin Q, Willis WD (2001) Nitric oxide synthase in spinal cord central sensitization following intradermal injection of capsaicin. Pain 94: 47–58. 11576744 10.1016/S0304-3959(01)00340-2

[8] Ernst AF, Wu HH, El-Fakahany EE, McLoon SC (1999) NMDA receptor-mediated refinement of a transient retinotectal projection during development requires nitric oxide. J Neurosci 19: 229–235. 9870953 10.1523/JNEUROSCI.19-01-00229.1999 PMC6782382

[9] Forstermann U, Mulsch A, Bohme E, Busse R (1986) Stimulation of soluble guanylate cyclase by an acetylcholine-induced endothelium-derived factor from rabbit and canine arteries. Circ Res 58: 531–538. 2870826 10.1161/01.res.58.4.531

[10] Ignarro LJ, Harbison RG, Wood KS, Kadowitz PJ (1986) Activation of purified soluble guanylate cyclase by endothelium-derived relaxing factor from intrapulmonary artery and vein: stimulation by acetylcholine, bradykinin and arachidonic acid. J Pharmacol Exp Ther 237: 893–900. 2872327

[11] Ignarro LJ, Adams JB, Horwitz PM, Wood KS (1986) Activation of soluble guanylate cyclase by NO-hemoproteins involves NO-heme exchange. Comparison of heme-containing and heme-deficient enzyme forms. J Biol Chem 261: 4997–5002. 2870064

[12] Bredt DS, Glatt CE, Hwang PM, Fotuhi M, Dawson TM, et al (1991) Nitric oxide synthase protein and mRNA are discretely localized in neuronal populations of the mammalian CNS together with NADPH diaphorase. Neuron 7: 615–624. 1718335 10.1016/0896-6273(91)90374-9

[13] Judas M, Sestan N, Kostovic I (1999) Nitrinergic neurons in the developing and adult human telencephalon: transient and permanent patterns of expression in comparison to other mammals. Microsc Res Tech 45: 401–419. 10402267 10.1002/(SICI)1097-0029(19990615)45:6<401::AID-JEMT7>3.0.CO;2-Q

[14] Zhang YT, Zhang DL, Cao YL, Zhao BL (2002) Developmental expression and activity variation of nitric oxide synthase in the brain of golden hamster. Brain Res Bull 58: 385–389. 12183015 10.1016/s0361-9230(02)00808-0

[15] Gotti S, Sica M, Viglietti-Panzica C, Panzica G (2005) Distribution of nitric oxide synthase immunoreactivity in the mouse brain. Microsc Res Tech 68: 13–35. 16208717 10.1002/jemt.20219

[16] Oermann E, Bidmon HJ, Mayer B, Zilles K (1999) Differential maturational patterns of nitric oxide synthase-I and NADPH diaphorase in functionally distinct cortical areas of the mouse cerebral cortex. Anat Embryol (Berl) 200: 27–41. 10395003 10.1007/s004290050256

[17] Chung YH, Joo KM, Lee YJ, Shin DH, Cha CI (2004) Postnatal development and age-related changes in the distribution of nitric oxide synthase-immunoreactive neurons in the visual system of rats. Neurosci Lett 360: 1–4. 15082164 10.1016/j.neulet.2004.01.002

[18] Terada H, Nagai T, Kimura H, Kitahama K, Okada S (1996) Distribution of nitric oxide synthase-immunoreactive neurons in fetal rat brains at embryonic day 15 and day 19. J Chem Neuroanat 10: 273–278. 8811417 10.1016/0891-0618(96)00141-x

[19] Terada H, Nagai T, Okada S, Kimura H, Kitahama K (2001) Ontogenesis of neurons immunoreactive for nitric oxide synthase in rat forebrain and midbrain. Brain Res Dev Brain Res 128: 121–137. 11412898 10.1016/s0165-3806(01)00162-6

[20] Iwase K, Iyama K, Akagi K, Yano S, Fukunaga K, et al (1998) Precise distribution of neuronal nitric oxide synthase mRNA in the rat brain revealed by non-radioisotopic in situ hybridization. Brain Res Dev Brain Res 53: 1–12. 10.1016/s0169-328x(97)00139-3 9473561

[21] Poon KL, Richardson M, Lam CS, Khoo HE, Korzh V (2003) Expression pattern of neuronal nitric oxide synthase in embryonic zebrafish. Gene Expr Patterns 3: 463–466. 12915313 10.1016/s1567-133x(03)00063-2

[22] Holmqvist B, Ellingsen B, Forsell J, Zhdanova I, Alm P (2004) The early ontogeny of neuronal nitric oxide synthase systems in the zebrafish. J Exp Biol 207: 923–935. 14766951 10.1242/jeb.00845

[23] Bruning G, Mayer B (1996) Prenatal development of nitric oxide synthase in the mouse spinal cord. Neurosci Lett 202: 189–192. 8848263 10.1016/0304-3940(95)12239-7

[24] Hess DT, Patterson SI, Smith DS, Skene JH (1993) Neuronal growth cone collapse and inhibition of protein fatty acylation by nitric oxide. Nature 366: 562–565. 8255294 10.1038/366562a0

[25] Hindley S, Juurlink BH, Gysbers JW, Middlemiss PJ, Herman MA, et al (1997) Nitric oxide donors enhance neurotrophin-induced neurite outgrowth through a cGMP-dependent mechanism. J Neurosci Res 47: 427–439. 9057136

[26] Gibbs SM, Becker A, Hardy RW, Truman JW (2001) Soluble guanylate cyclase is required during development for visual system function in Drosophila. J Neurosci 21: 7705–7714. 11567060 10.1523/JNEUROSCI.21-19-07705.2001 PMC6762879

[27] Gibbs SM, Truman JW (1998) Nitric oxide and cyclic GMP regulate retinal patterning in the optic lobe of Drosophila. Neuron 20: 83–93. 9459444 10.1016/s0896-6273(00)80436-5

[28] Tojima T, Itofusa R, Kamiguchi H (2009) The nitric oxide-cGMP pathway controls the directional polarity of growth cone guidance via modulating cytosolic Ca2+ signals. J Neurosci 29: 7886–7897. 19535600 10.1523/JNEUROSCI.0087-09.2009 PMC6665622

[29] Yamazaki M, Chiba K, Mohri T (2006) Differences in neuritogenic response to nitric oxide in PC12 and PC12h cells. Neurosci Lett 393: 222–225. 16239071 10.1016/j.neulet.2005.09.068

[30] Yamada RX, Matsuki N, Ikegaya Y (2006) Nitric oxide/cyclic guanosine monophosphate-mediated growth cone collapse of dentate granule cells. Neuroreport 17: 661–665. 16603931 10.1097/00001756-200604240-00021

[31] Ernst AF, Gallo G, Letourneau PC, McLoon SC (2000) Stabilization of growing retinal axons by the combined signaling of nitric oxide and brain-derived neurotrophic factor. J Neurosci 20: 1458–1469. 10662836 10.1523/JNEUROSCI.20-04-01458.2000 PMC6772364

[32] Tornieri K, Rehder V (2007) Nitric oxide release from a single cell affects filopodial motility on growth cones of neighboring neurons. Dev Neurobiol 67: 1932–1943. 17874460 10.1002/dneu.20572

[33] Welshhans K, Rehder V (2005) Local activation of the nitric oxide/cyclic guanosine monophosphate pathway in growth cones regulates filopodial length via protein kinase G, cyclic ADP ribose and intracellular Ca2+ release. Eur J Neurosci 22: 3006–3016. 16367767 10.1111/j.1460-9568.2005.04490.x

[34] Welshhans K, Rehder V (2007) Nitric oxide regulates growth cone filopodial dynamics via ryanodine receptor-mediated calcium release. Eur J Neurosci 26: 1537–1547. 17714493 10.1111/j.1460-9568.2007.05768.x

[35] Trimm KR, Rehder V (2004) Nitric oxide acts as a slow-down and search signal in developing neurites. Eur J Neurosci 19: 809–818. 15009128 10.1111/j.0953-816x.2004.03182.x

[36] Van Wagenen S, Rehder V (1999) Regulation of neuronal growth cone filopodia by nitric oxide. J Neurobiol 39: 168–185. 10235672 10.1002/(sici)1097-4695(199905)39:2<168::aid-neu2>3.0.co;2-f

[37] Van Wagenen S, Rehder V (2001) Regulation of neuronal growth cone filopodia by nitric oxide depends on soluble guanylyl cyclase. J Neurobiol 46: 206–219. 11169506 10.1002/1097-4695(20010215)46:3<206::aid-neu1003>3.3.co;2-j

[38] Wu HH, Cork RJ, Huang PL, Shuman DL, Mize RR (2000) Refinement of the ipsilateral retinocollicular projection is disrupted in double endothelial and neuronal nitric oxide synthase gene knockout mice. Brain Res Dev Brain Res 120: 105–111. 10727738 10.1016/s0165-3806(99)00145-5

[39] Campello-Costa P, Fosse AM Jr, Ribeiro JC, Paes-De-Carvalho R, Serfaty CA (2000) Acute blockade of nitric oxide synthesis induces disorganization and amplifies lesion-induced plasticity in the rat retinotectal projection. J Neurobiol 44: 371–381. 10945893 10.1002/1097-4695(20000915)44:4<371::aid-neu1>3.0.co;2-x

[40] Wang T, Xie Z, Lu B (1995) Nitric oxide mediates activity-dependent synaptic suppression at developing neuromuscular synapses. Nature 374: 262–266. 7885445 10.1038/374262a0

[41] Bradley S, Tossell K, Lockley R, McDearmid J (2010) Nitric oxide synthase regulates morphogenesis of zebrafish spinal cord motoneurons. J Neurosci 30: 16818–16831. 21159953 10.1523/JNEUROSCI.4456-10.2010 PMC6634927

[42] Westerfield M (1994) The Zebrafish Book: a Guide to the Laboratory Use of the Zebrafish (Brachydanio rerio): Eugene, OR: University of Oregon Press.

[43] Kimmel CB, Ballard WW, Kimmel SR, Ullmann B, Schilling TF (1995) Stages of embryonic development of the zebrafish. Dev Dyn 203: 253–310. 8589427 10.1002/aja.1002030302

[44] Luna VM, Brehm P (2006) An electrically coupled network of skeletal muscle in zebrafish distributes synaptic current. J Gen Physiol 128: 89–102. 16801383 10.1085/jgp.200609501 PMC2151551

[45] del Castillo J, Escalona de Motta G (1978) A new method for excitation-contraction uncoupling in frog skeletal muscle. J Cell Biol 78: 782–784. 568143 10.1083/jcb.78.3.782 PMC2110188

[46] Buss RR, Drapeau P (2002) Activation of embryonic red and white muscle fibers during fictive swimming in the developing zebrafish. J Neurophysiol 87: 1244–1251. 11877498 10.1152/jn.00659.2001

[47] Herberich E, Sikorski J, Hothorn T (2010) A robust procedure for comparing multiple means under heteroscedasticity in unbalanced designs. PloS One 5: e9788. 20360960 10.1371/journal.pone.0009788 PMC2847912

[48] Panzer JA, Gibbs SM, Dosch R, Wagner D, Mullins MC, et al (2005) Neuromuscular synaptogenesis in wild-type and mutant zebrafish. Dev Biol 285: 340–357. 16102744 10.1016/j.ydbio.2005.06.027

[49] van Raamsdonk W, Pool CW, te Kronnie G (1978) Differentiation of muscle fiber types in the teleost Brachydanio rerio. Anat Embryol (Berl) 153: 137–155. 677468 10.1007/BF00343370

[50] Buss RR, Drapeau P (2000) Physiological properties of zebrafish embryonic red and white muscle fibers during early development. J Neurophysiol 84: 1545–1557. 10980026 10.1152/jn.2000.84.3.1545

[51] Buckingham SD, Ali DW (2004) Sodium and potassium currents of larval zebrafish muscle fibres. J Exp Biol 207: 841–852. 14747415 10.1242/jeb.00839

[52] Flanagan-Steet H, Fox MA, Meyer D, Sanes JR (2005) Neuromuscular synapses can form in vivo by incorporation of initially aneural postsynaptic specializations. Development 132: 4471–4481. 16162647 10.1242/dev.02044

[53] Jing L, Lefebvre JL, Gordon LR, Granato M (2009) Wnt signals organize synaptic prepattern and axon guidance through the zebrafish unplugged/MuSK receptor. Neuron 61: 721–733. 19285469 10.1016/j.neuron.2008.12.025 PMC2671566

[54] Liu DW, Westerfield M (1992) Clustering of muscle acetylcholine receptors requires motoneurons in live embryos, but not in cell culture. J Neurosci 12: 1859–1866. 1315852 10.1523/JNEUROSCI.12-05-01859.1992 PMC6575886

[55] Sylvain NJ, Brewster DL, Ali DW (2011) Embryonic ethanol exposure alters synaptic properties at zebrafish neuromuscular junctions. Neurotoxicol Teratol 33: 313–321. 21167937 10.1016/j.ntt.2010.12.001

[56] Drapeau P, Buss RR, Ali DW, Legendre P, Rotundo RL (2001) Limits to the development of fast neuromuscular transmission in zebrafish. J Neurophysiol 86: 2951–2956. 11731551 10.1152/jn.2001.86.6.2951

[57] Nguyen PV, Aniksztejn L, Catarsi S, Drapeau P (1999) Maturation of neuromuscular transmission during early development in zebrafish. J Neurophysiol 81: 2852–2861. 10368402 10.1152/jn.1999.81.6.2852

[58] Kullberg RW, Mikelberg FS, Cohen MW (1980) Contribution of Cholinesterase to Developmental Decreases in the Time Course of Synaptic Potentials at an Amphibian Neuromuscular-Junction. Dev Biol 75: 255–267. 6245982 10.1016/0012-1606(80)90161-x

[59] Mongeon R, Walogorsky M, Urban J, Mandel G, Ono F, et al (2011) An acetylcholine receptor lacking both gamma and epsilon subunits mediates transmission in zebrafish slow muscle synapses. J Gen Physiol 138: 353–366. 21844221 10.1085/jgp.201110649 PMC3171075

[60] Mishina M, Takai T, Imoto K, Noda M, Takahashi T, et al (1986) Molecular Distinction between Fetal and Adult Forms of Muscle Acetylcholine-Receptor. Nature 321: 406–411. 2423878 10.1038/321406a0

[61] Naranjo D, Brehm P (1993) Modal shifts in acetylcholine receptor channel gating confer subunit-dependent desensitization. Science 260: 1811–1814. 8511590 10.1126/science.8511590

[62] Missias AC, Chu GC, Klocke BJ, Sanes JR, Merlie JP (1996) Maturation of the acetylcholine receptor in skeletal muscle: Regulation of the AChR gamma-to-epsilon switch. Dev Biol 179: 223–238. 8873766 10.1006/dbio.1996.0253

[63] Gu Y, Hall ZW (1988) Immunological evidence for a change in subunits of the acetylcholine receptor in developing and denervated rat muscle. Neuron 1: 117–125. 3272161 10.1016/0896-6273(88)90195-x

[64] Kyriakatos A, Molinari M, Mahmood R, Grillner S, Sillar KT, et al (2009) Nitric oxide potentiation of locomotor activity in the spinal cord of the lamprey. J Neurosci 29: 13283–13291. 19846716 10.1523/JNEUROSCI.3069-09.2009 PMC6665181

[65] McLean DL, Sillar KT (2004) Metamodulation of a spinal locomotor network by nitric oxide. J Neurosci 24: 9561–9571. 15509743 10.1523/JNEUROSCI.1817-04.2004 PMC6730165

[66] Luna VM, Wang M, Ono F, Gleason MR, Dallman JE, et al (2004) Persistent electrical coupling and locomotory dysfunction in the zebrafish mutant shocked. J Neurophysiol 92: 2003–2009. 15201312 10.1152/jn.00454.2004

[67] Katz B, Miledi R (1965) The Measurement of Synaptic Delay, and the Time Course of Acetylcholine Release at the Neuromuscular Junction. Proc R Soc Lond B Biol Sci 161: 483–495. 14278409 10.1098/rspb.1965.0016

[68] Wen H, Brehm P (2005) Paired motor neuron-muscle recordings in zebrafish test the receptor blockade model for shaping synaptic current. J Neurosci 25: 8104–8111. 16135768 10.1523/JNEUROSCI.2611-05.2005 PMC6725451

[69] Samigullin D, Bukharaeva EA, Nikolsky E, Adamek S, Vyskocil F (2003) Long release latencies are increased by acetylcholine at frog endplate. Physiol Res 52: 475–480. 12899661

[70] Bukcharaeva EA, Kim KC, Moravec J, Nikolsky EE, Vyskocil F (1999) Noradrenaline synchronizes evoked quantal release at frog neuromuscular junctions. J Physiol 517: 879–888. 10358126 10.1111/j.1469-7793.1999.0879s.x PMC2269380

[71] Datyner NB, Gage PW (1980) Phasic Secretion of Acetylcholine at a Mammalian Neuromuscular-Junction. J Physiol 303: 299–314. 6253620 10.1113/jphysiol.1980.sp013286 PMC1282892

[72] Ribera J, Marsal J, Casanovas A, Hukkanen M, Tarabal O, et al (1998) Nitric oxide synthase in rat neuromuscular junctions and in nerve terminals of Torpedo electric organ: Its role as regulator of acetylcholine release. J Neurosci Res 51: 90–102. 9452313 10.1002/(SICI)1097-4547(19980101)51:1<90::AID-JNR10>3.0.CO;2-C

[73] Tricoire L, Vitalis T (2012) Neuronal nitric oxide synthase expressing neurons: a journey from birth to neuronal circuits. Front Neural Circuits 6: 82. 23227003 10.3389/fncir.2012.00082 PMC3514612

[74] Saint-Amant L, Drapeau P (1998) Time course of the development of motor behaviors in the zebrafish embryo. J Neurobiol 37: 622–632. 9858263 10.1002/(sici)1097-4695(199812)37:4<622::aid-neu10>3.0.co;2-s

[75] McLean DL, Masino MA, Koh IY, Lindquist WB, Fetcho JR (2008) Continuous shifts in the active set of spinal interneurons during changes in locomotor speed. Nat Neuro 11: 1419–1429. 10.1038/nn.2225 PMC2735137 18997790

[76] McLean DL, Fan J, Higashijima S, Hale ME, Fetcho JR (2007) A topographic map of recruitment in spinal cord. Nature 446: 71–75. 17330042 10.1038/nature05588

[77] Bhatt DH, McLean DL, Hale ME, Fetcho JR (2007) Grading movement strength by changes in firing intensity versus recruitment of spinal interneurons. Neuron 53: 91–102. 17196533 10.1016/j.neuron.2006.11.011

[78] Menelaou E, McLean DL (2012) A gradient in endogenous rhythmicity and oscillatory drive matches recruitment order in an axial motor pool. J Neurosci 32: 10925–10939. 22875927 10.1523/JNEUROSCI.1809-12.2012 PMC3428065

[79] Ausborn J, Mahmood R, El Manira A (2012) Decoding the rules of recruitment of excitatory interneurons in the adult zebrafish locomotor network. Proc Natl Acad Sci U S A 109: E3631–3639. 23236181 10.1073/pnas.1216256110 PMC3535644

[80] Gabriel JP, Ausborn J, Ampatzis K, Mahmood R, Eklof-Ljunggren E, et al (2011) Principles governing recruitment of motoneurons during swimming in zebrafish. Nat Neurosci 14: 93–99. 21113162 10.1038/nn.2704

